# Imbalances in the Study of the Relationship between Leisure and Self-Esteem: A Systematic Review

**DOI:** 10.3390/ijerph17155555

**Published:** 2020-07-31

**Authors:** Nuria Codina, Teresa Freire

**Affiliations:** 1 Department of Social Psychology and Quantitative Psychology, University of Barcelona, 08035 Barcelona, Spain; 2 Department of Applied Psychology, School of Psychology, University of Minho, 4710-057 Braga, Portugal; tfreire@psi.uminho.pt

**Keywords:** leisure, self-esteem, systematic review, subjective analysis, objective analysis

## Abstract

This systematic review offers a comprehensive examination of the relationship between leisure and self-esteem. The different perspectives were analyzed according to a framework that includes the different approaches for defining and measuring leisure, and a similar one was proposed for self-esteem. Articles indexed in the Web of Science (WoS) up to the end of 2018 were reviewed, specifically those that contained the keywords “leisure”, “self-esteem” or “self esteem” anywhere in the manuscript. Articles that did not present the qualitative or quantitative instruments needed to evaluate leisure or self-esteem were excluded. A total of 49 articles included the final quantitative synthesis. The overall findings showed that the prevailing methodology was objective (external). As regards content, the following combinations predominated: the behavioral approach to leisure with the unidimensional approach to self-esteem and the experiential approach to leisure with the unidimensional approach to self-esteem. Less studies were observed with the combination of mixed approaches and more comprehensive analyses: the behavioral-experiential combined with the multidimensional. To conclude, this study shows there is a demand for further empirical studies that explore the relationships between leisure and self-esteem. It also identified which approaches are most desirable to expand our understanding of the relationships between leisure and self-esteem.

## 1. Introduction

The relationship between leisure and self-esteem does not seem open to doubt, with many specialists insisting on its validity in recent decades (see, among others, [1,2,3]). It is a relationship of recognized importance where leisure is understood to be a creator of opportunities that have a resultant impact on self-esteem [4,5]. Leisure provides opportunities for the person enhance and to engage in freely chosen life events and experiences [6]—and this is related to the wellbeing of the person, since it refers to how “people feel good about themselves and feel that they are lovable and worthwhile people” [7] (p. 181).

However, some research has revealed distinctions in this relationship that invite a review of this field of study. For example, it has been found that an increase in self-esteem is associated with the practice of leisure activities that are significant for the person or activities where social support from others is perceived [8,9,10,11], while the practice of sedentary activities or activities whose difficulty causes stress is associated with a loss of self-esteem [12,13,14]. Furthermore, self-esteem is seen to be nourished by both leisure and non-leisure, to the point that work is more important than leisure for some people [15,16]. Despite these distinctions in the relationship between leisure and self-esteem, there are no articles that discuss them or further explore new findings. At the same time, no studies provide a theoretical reflection and guidance as regards which different aspects of leisure and self-esteem should be evaluated when studying their relationship. In the same vein, research is needed to assess what knowledge can be gathered by adopting different reference points and conceptual and methodological approaches. In other words, there is no research that makes a firm commitment to introducing the study of leisure into the exploration of the dynamics of self-esteem and which thereby facilitates the investigation and comprehensive understanding of this relationship.

Given these observations, this paper evaluates the current studies on leisure and self-esteem by analyzing the contribution made by conceptual and methodological approaches that serve to study both topics. Then, we draw on these approaches to present a systematic review of the scientific publications that empirically analyze the relationship between leisure and self-esteem. With this study, we reveal the possibilities and limitations of the research carried out so far and show what is needed to make progress towards a comprehensive understanding of the relationship between leisure and self-esteem.

### 1.1. Analyzing Leisure from a Plural Perspective

Although leisure is commonly understood from a lay perspective, among scientific literature a diversity of leisure definitions exists making difficult a unique definition of leisure [17]. Despite this, researchers have been trying to converge characterizing leisure, highlighting relevant dimensions and categories in order to understand the plurality of perspectives based such a multidisciplinary topic. According to this and apart from the different scientific lens, leisure can be characterized as a life time commonly associated to free time, as activities, as experiences, and/or as contexts, underlying the coexistence and relationships with the elements in one’s environment. Based on this plurality of perspectives, authors tend to agree that each one of them alone is insufficient, and thus, an integral/holistic analysis of the phenomenon should integrate all the valued aspects.

The comprehensive understanding of leisure, which counts on a plural, solid, well-grounded body of knowledge, also contemplates different approaches to its study, that is, an epistemological and methodological pluralism as regards the differences of conceptions about what is considered legitimate. Advocating in favor of a comprehensive study of leisure are Kleiber, Walker and Mannell [18,19], who developed a useful research framework based on different research approaches adopted to define and measure leisure. Specifically, their model makes it possible to organize, evaluate and orientate comprehensive research into the leisure phenomenon. The framework distinguishes two approaches to analysis (previously specified, among others, by Neulinger, Ellis and Witt, or Lawton [20,21,22]): objective leisure and subjective leisure. The objective approach focuses on the assessment of activities, settings or time periods; and the subjective approach contemplates certain types of meanings, experiences and satisfied needs, which derive from the significance of the activity and not from the activity itself. In addition to these, they distinguish two perspectives from which the phenomenon is defined and operationalized, based on the researcher’s viewpoint (external definitional) or the participant’s viewpoint (internal definitional). As a result of these different approaches to “what” and “how” leisure is analyzed, the authors plotted a two by-two matrix (Table 1.).

Four sets of data are obtained by combining the two perspectives of the object of study (objective or subjective behavior) and the two types of agent who structure the information (internal or external informant). We now briefly describe each of them.

Behavioral-Observer: The practice of an activity is recorded in a setting or time period. The researchers define leisure and non-leisure.

Experiential-Observer: Leisure is valued in terms of experience, satisfaction or meaning associated with involvement. The researchers define leisure and non-leisure.

Behavioral-Participant: The practice of an activity is recorded in a setting or time period. The participants define leisure and non-leisure.

Experiential-Participant: Leisure is valued in terms of experience, satisfaction or meaning associated with involvement. The participants define leisure and non-leisure.

As pointed out above, research into the phenomenon requires these four complementary approaches if the goal is to try to obtain a full picture or an understanding of leisure in all its complexity. In effect, and as stated by various authors, the divergences—between what researchers and participants consider leisure or between what defines the leisure experience for researchers and participants—enrich the corpus of knowledge because the researchers are obliged to explain and investigate the said divergences [23,24,25,26]. In this respect, Shaw [27] affirms that if leisure is only studied from the researcher’s standpoint, leisure that is significant for people may be neglected; and Godbey and Song [28] observe that the fact of having disregarded laypeople’s perspectives has hampered the growth of knowledge regarding the phenomenon of leisure in non-Western cultures. Consequently, the ideal solution is to combine the different approaches in the same study. However, this is not an easy task. One good alternative is that with respect to a topic, while individually different research projects may adopt one of the four approaches, all four could be taken into consideration, inasmuch as is possible, in the whole set of studies. Ultimately, this model is useful for evaluating to what extent, in the study of the relationship between leisure and self-esteem, leisure is analyzed comprehensively, maintaining a certain balance between different approaches or focusing on certain perspectives and research strategies.

### 1.2. Analyzing Self-Esteem in Its Dimensions

The above framework guides the study of leisure and we draw on it to propose a similar one for the comprehensive study of self-esteem. In general terms, the scientific literature understands self-esteem as a self-assessment–which generates feelings and emotions towards oneself that may range from approval to disapproval [29,30,31]. The APA [32] (p. 995) defines it as “the degree to which the qualities and characteristics contained in one’s self-concept are perceived to be positive”. But before detailing our proposal, we will briefly consider the approaches adopted to study self-esteem.

The concept of self-esteem has become increasingly popular in psychological and psychosocial studies in recent decades [33,34,35,36]. As pointed out by Habrat [37], this popularity has made it a household word thanks to the literature with limited scientific import that responds to certain specific problems [37,38,39]. On the other hand, self-esteem has also been thoroughly researched thanks to its popularity, which has worked in favor of its theoretical and empirical operationalization and shown that it is a construct of a complex nature with diverse dimensions.

**Figure 1:**
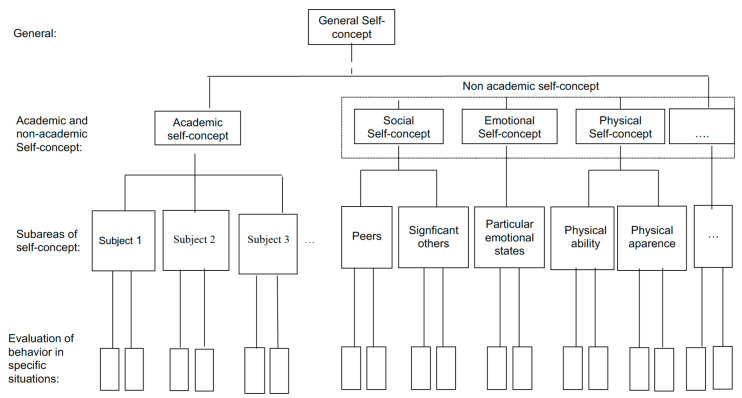
Representation of a multidimensional, hierarchical model (Adapted from: Shavelson, Hubner and Stanton [41], p. 413; original version copyright is the American Educational Research Association).

The concept of self-esteem, in a more operative sense, has been conceptualized as both a unidimensional and a multidimensional phenomenon. Initially, self-esteem was conceived as a global construct, referred to in terms of a unidimensional model or global self-esteem. In this case, self-esteem is understood to be a positive or negative general attitude or feeling towards oneself [40]. Subsequently, the multidimensional conception of the self-emerged and gathered impetus thanks to the model proposed by Shavelson, Hubner and Stanton [41] (Figure 1). From this perspective, which also includes global self-esteem, it is understood that self-esteem derives from experiences in different areas of a person’s life, also called domains, dimensions or components, which are integrated by specific competences organized in a hierarchical manner. In other words, although the terms self-concept and self-esteem are sometimes used interchangeably, the latter is strictly limited to the evaluative aspect of self-concept, which derives from the conscious perceptions about oneself in different domains [32].

Consequently, given that a person’s life is affected by different areas of competence and relationships (family, school, work, social, physical, out-of-school, etc.), it is understood that the person has a specific self-image in each one of these areas and that the global self-image derives from the whole set [41,42,43]. Therefore, specific self-esteem is a better predictor of a specific behavior, and global self-esteem, of general wellbeing [33,35,37]. The versatility of the multidimensionality of the self, and above all its potential to explain and intervene, has promoted the development of different multidimensional models, which incorporate specific dimensions and subareas in order to identify what and how certain areas affect self-esteem (for example, the models developed by [44,45,46,47]).

In a more practical sense, the application of multidimensional models has shown, for example, that higher global self-esteem is related to physical self-esteem, which derives from participation in physical activities [48,49]. Thus, it would seem necessary to adopt a multidimensional perspective of the construct to discover the details of the relationship between leisure activities and self-esteem. If the work of Campbell, Eisner and Riggs [50] is adapted to the field of leisure, this premise can be supported by assumptions such as the following: If a person is very good at sport and bad at relationships, we will find this difference if we take a multidimensional approach, but we will not find this difference reflected in a global or one-dimensional approach that includes the dimensions of sport activities and relationships. On the other hand, the multidimensional approach also has its limitations. For example, the dimension of leisure and its associated attributes are not clearly delimited in multidimensional models and working with these models is much more costly in terms of the time required of participants. Furthermore, it is extremely difficult to standardize the instruments to ensure they are valid for participants with different ages and from different countries or cultures [51]. Having ascertained the possibilities and limitations of the multidimensional model, a successful research model to better understand the complexity of self-esteem would be one that combines standardized unidimensional and multidimensional approaches with qualitative methodological approaches or one that opts for a qualitative multidimensional approach [37,39,52,53,54,55].

### 1.3. Analyzing the Plurality of the Sources of Self-Esteem

Having detailed the conceptual and methodological considerations about self-esteem, we propose a framework that contemplates, on the one hand, the unidimensional and multidimensional analysis of self-esteem and, on the other hand, the analysis of the person’s self-evaluations, which can be carried out according to the content proposed by the person under study (internal) or the researcher (external) (Table 2). Therefore, the framework is structured as follows:

Four sets of data are obtained by combining the two concepts related to the object of study (unidimensional or multidimensional) and the two types of agent who structure the information (internal or external informant).

Unidimensional-observer: Self-esteem is understood to be a global self-evaluation. The researcher determines the specific questions that he/she considers as defining general self-esteem.

Multidimensional-observer: Self-esteem is understood to be the result of specific evaluations in different areas of everyday life. The researcher determines the dimensions and the specific questions that he/she considers as defining each specific dimension and general self-esteem.

Unidimensional-participant: Self-esteem is understood to be a global self-evaluation. The participant evaluates himself/herself on the basis of specific questions that, in his/her opinion, define general self-esteem.

Multidimensional-participant: Self-esteem is understood to be the result of specific evaluations in different areas of everyday life. The participant values himself/herself according to those dimensions that he/she considers relevant.

This framework for the analysis of self-esteem is based in the model proposed by Kleiber, Walker and Mannell [16] and also serves to assess the plurality of approaches to the study of the phenomenon. Then again, as regards the necessary complementarity of perspectives noted in the case of leisure, such complementarity in self-esteem emerges in the participant’s and the researcher’s perspectives, and the differences provide information that generates new knowledge [37,39,56]. On these lines, for example, it has been observed that the results obtained from unstructured and semi-structured interviews question the meaning of certain sections of the recommended scales. In particular, Schwan, Fallonbar and Milne, 2018 [57], using semi-structured interviews, found that what makes the homeless have positive feelings and emotions towards themselves is the creation of a product, the achievement of a goal, the discovery of themselves and so on—aspects that are not included in the recommended scales for research on self-esteem. However, as the authors themselves point out, these interviews would have to be complemented by the observer approach to more constructively enrich the body of knowledge. As to the unidimensional and multidimensional perspectives, their complementarity is relative because one of them, the multidimensional perspective, does not renounce the evaluation of global self-esteem. In this case, new knowledge emerges thanks to the precision with which the sources of self-esteem are evaluated, i.e., activities, contexts, relationships, etc., that affect self-esteem [35,36,37]. However, as pointed out above, the multidimensional approach has its limitations, and one in particular in the case of the study of leisure is the fact of not having worked seriously to ensure leisure is fully incorporated into the study of self-esteem.

### 1.4. About This Study

After delimiting the two concepts, we went on to explore the approaches used to study the relationship between leisure and self-esteem. The goal was to map the combination of approaches, identify the most and least popular, and discover the possibilities, limitations and challenges that this line of work presents with respect to the goal of developing a comprehensive body of knowledge. With this in mind, we carried out a systematic review of the published works that relate leisure to self-esteem. Specifically, we expected to find that the relationship between leisure and self-esteem had been analyzed and explained by combining the four approaches to analysis available in each case. In other words, as far as leisure is concerned, we expected this to have been studied from both objective and subjective perspectives as well as observational and participant ones, and regarding self-esteem, we expected this to have been studied from the unidimensional and multidimensional perspectives as well as observational and participant ones.

## 2. Materials and Methods

This review followed the 27 statements of the PRISMA guidelines for systematic reviews.

The search was carried out using the WoS database and concluded on 10 January 2020. In order to review complete years and due to the delay in updating the databases, the period reviewed finished on 31 December 2018 (any articles published and added to this database after that date were not included). The WoS Core Collection database was searched via the electronic interface and the Boolean search command used was “leisure” AND “self-esteem” OR “self-esteem”. Two reviewers judged independently whether the studies met the inclusion criteria. Any disagreements about whether or not to include studies were resolved between the authors. No third party was consulted. The criteria applied when considering studies for possible review were as follows.

The inclusion criteria were met by:

1. Articles published up to the end of December 2018.
2. Articles published in peer review journals indexed in the WoS Core Collection.
3. Articles containing the keywords “leisure” and “self-esteem” or “self esteem” anywhere in the manuscript.

The exclusion criteria were met by:

1. Articles without “leisure” and “self-esteem” or “self esteem” in the title, keywords or abstract.
2. Articles that did not include empirical research.
3. Articles without qualitative or quantitative instruments needed to evaluate self-esteem.

**Figure 2:**
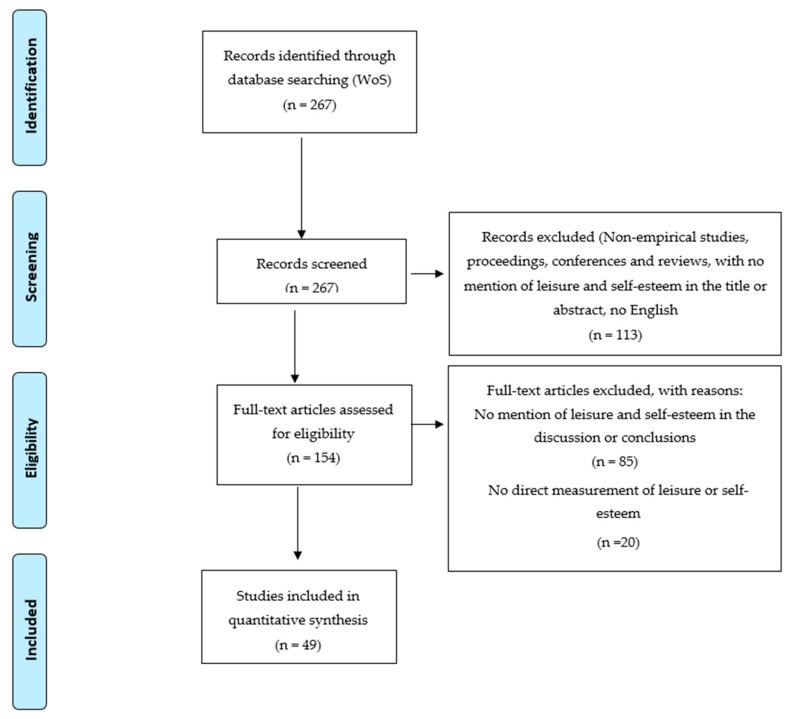
Selection of eligible articles.

After considering all the search results, we organized the information according to the above criteria. A total of 49 articles were finally considered eligible for review, as shown in Figure 2.

## 3. Results

According to the WoS records, the total number of studies in which the relationship between leisure and self-esteem assumes some centrality and is empirically analyzed was 49 (Table 3). Regarding variable leisure and in line with the framework developed by Kleiber et al. [18], a total of 18 out of these 49 articles (Table 4) were included in the behavioral-observer category, and 12 in the experiential-observer category, highlighting an external definitional point in both cases. When looking for an internal definitional point, three articles were included in the behavioral-participant category, two articles in the experiential-participant category and one article in the observer-experiential participant category. Thus, and according to the type of phenomena, 21 articles were associated with behavioral phenomena and 14 with experiential phenomena. In addition, 13 articles were identified that form part of three categories (behavioral-observer, experiential-observer and behavioral-experiential participant). On the other hand, as far as the observer-participant approach is concerned, 30 papers valued leisure in terms of an observer approach, i.e., leisure studies where criteria or variables selected by the observer predominate. Finally, a minority consisting of just five articles analyzed leisure from the participant approach or the internal perspective and one combined the modalities of the observer and participant approaches. As regards the variable of self-esteem and its dimensionality, 43 articles conceptualized self-esteem as a unidimensional construct and six, as a multidimensional construct (Table 4). With respect of the observer or participant approach, only one article used the participant unidimensional approach.

As far as the joint study of leisure and self-esteem is concerned, on considering the different approaches to the study of leisure and the unidimensional and multidimensional approaches to the study of self-esteem, we found that in the case of behavioral approaches, whether behavioral-observer or behavioral-participant (or a combination of both), self-esteem is studied as a unidimensional phenomenon (22). This same tendency in the study of self-esteem is observed when the approach to the study of leisure is experiential (14). As reported in Table 4, multidimensional analyzes of self-esteem were found in papers where the study of leisure combines behavioral and experiential approaches. In effect, almost half of the studies (6) in this case analyzed the self from a multidimensional perspective (it was analyzed as unidimensional in the rest). Considering the studies with minority combinations, it is observed that only on one occasion—the sole case identified in this review—was self-esteem studied from an internal perspective, and leisure was analyzed using a behavioral-experiential-internal approach on this occasion.

## 4. Discussion

Although the scientific literature links leisure to self-esteem, the scientific research that strongly supports this relationship is limited. In our research, despite finding 154 articles that give the terms leisure and self-esteem a certain amount of importance—including them in the title, abstract or keywords, only in 49 cases were efforts made to empirically register both realities, the first in 1992 [102,103]. In other words, the relationship between leisure and self-esteem has limited significance in over a third of these articles: It is not empirically studied, nor is it mentioned in the discussion or conclusions. Consequently, if the number of publications in which these concepts are central is compared with the number of those that empirically investigate them, it can be seen that there are relatively few studies whose goal was to generate new knowledge in this field with a proven foundation. Thus, with respect to the assumption of the unquestionable relationship between the two concepts, a certain amount of liberty appears to have been taken by defending this idea without presenting any evidence, which does not favor the advance of knowledge.

Among those studies where the relationship between leisure and self-esteem is given particular attention, certain approaches take precedence, while others are minority or non-existent. Specifically, leisure is analyzed for the most part by applying observational methodologies in both the behavioral and experiential approaches. The participant approaches are in the minority, especially when conjugated with experiential approaches. As regards the study of self-esteem, the one-dimensional approach combined with observational methodologies predominates. This trend is also the predominant one in the study of self-esteem in other contexts and settings; a trend, to clarify the point, that uses the Rosemberg self-esteem scale as the main reference [37,38,39].

When the diversity of approaches in the investigation of both phenomena is compared, we see that leisure is the more plural field. Nonetheless, and as previously affirmed by Roberts [104], the prevalence of the behavioral-observational approach can be understood as an example of why leisure studies are not making as much progress as would be desirable. A main limitation is the search for a theory that explains leisure while considering that only one of the available theories is going to be correct and ignoring the fact that the different theories are complementary.

In the combination of approaches to the joint study of leisure and self-esteem, the dominant ones are observational (behavioral or experiential), in the case of leisure, and unidimensional-observational, in the case of self-esteem, followed by those that combine the behavioral and experiential observation of leisure with the unidimensional and multidimensional observation of self-esteem. Therefore, there is very limited pluralism regarding the way of collecting information. On the other hand, there is a relative degree of pluralism in the content of the studies; to be more precise, relative in the case of leisure but less so in the case of self-esteem. Consequently, given that the body of knowledge concerning the relationship between leisure and self-esteem is mainly nourished by observational approaches, the behaviors and other aspects experienced by the participants are not sufficiently valued. In effect, in the set of studies reviewed, only a limited number take into account people’s evaluations of their behaviors and leisure experiences and their relationship with global or specific self-esteem–and this is not a trivial matter. Precisely this internal approach can provide very valuable information regarding how leisure and self-esteem influence each other and how these variables affect people’s wellbeing. In this sense, one of the reviewed studies—by Schwan et al. [57] and featuring a behavioral-experiential-unidimensional participant approach—showed that artistic creation is understood by the participants as a survival strategy rather than a hobby. It is positively experienced due to the perception of agency rather than from satisfaction, and it is valued–depending fundamentally on the achievement of goals or targets and learning things about oneself, as a source of self-assessment, self-fulfillment, self-efficacy, happiness and wellbeing. Accordingly, the internal approach provides a valuable strategy for furthering the understanding of the relationships between leisure and self-esteem and the contributions of leisure and self-esteem to people’s wellbeing.

With regard to the aforesaid predominant observational approach, the research combinations reveal the following: The behavioral approach combined with the unidimensional approach associates leisure practices with a global perception of self-esteem, without managing to specify what experiential aspects of leisure are associated with self-esteem or whether other areas of everyday life have an impact on self-esteem. Consequently, there is no contrasted scientific support underpinning this combination of approaches to the relationship between leisure and self-esteem and its contribution to wellbeing. In fact, a leisure or non-leisure activity may be linked to self-esteem or to the perception of wellbeing, not by the activity itself but by the experiences associated with it. Furthermore, neither can this combination of approaches explain the contradictions regarding how leisure or a specific leisure behavior is related to an increase or not in self-esteem. The combination of the experiential and unidimensional approaches contemplates the evaluation of leisure and its relationship with global self-esteem. Therefore, the analysis is more precise in this case; it is the qualities of leisure (satisfaction, perception of competence, etc.) that are analyzed in relation to self-esteem. However, this does not explain what leisure behaviors are related to self-esteem. As for the somewhat more mixed approaches, those that combine the behavioral and experiential approaches with the unidimensional approach can identify which qualities of certain activities are associated with global self-esteem. Moreover, the most mixed methodologies found in this review—those that combine the behavioral and experiential approaches with the multidimensional approach—can serve to identify what activities and what qualities of the activities affect certain dimensions of self-esteem. In short, in the last case, which is the combination of observational approaches and found in the fewest studies, a more complete analysis of this relationship is carried out. It is the approach that best predicts the influence of specific behaviors on self-esteem. Nonetheless, all these approaches suffer from the limitation of starting out with the observer’s analysis and disregarding the experience of the subjects themselves, as explained above. It is also important to note that when the study of leisure combines the behavioral and experiential approaches, then self-esteem is considered from a multidimensional perspective in nearly half of the cases. A point that seems to indicate that when a more comprehensive approach to leisure is taken, the researchers are seeking a more exact understanding of the components that make up self-esteem.

The precedence that some approaches take over others could be justified by research tradition or by the resources available to obtain and/or analyze data. Regarding leisure, the potential of leisure practices as a promoter of change, adaptation and social transformation, for example, has led to the objective study of leisure from the perspective of the observer [105,106]. In the case of self-esteem, the premise of the role played by tradition and the available resources might be confirmed by the fact of the application (in two-thirds of the sample) of the self-esteem scale most widely used in the past and the easiest to apply: the Rosenberg scale [37,107]. On the other hand, the exploration of minority approaches might be inhibited by the limitations that we have highlighted regarding the application of standardized multidimensional models (application costs, standardization, etc.), the low popularity of participant approaches in the study of self-esteem and, as far as leisure studies are concerned, the fact of not having seriously integrated the challenge of studying self-esteem.

In a more practical sense, having applied the proposed frameworks to assess the study of leisure and self-esteem, we note among other limitations in this body of knowledge that there is a lack of information concerning the following: which dimensions of self-esteem are most benefited by leisure practices and experiences; which kinds of leisure not contemplated by the research contribute to self-esteem; the differences in the relationship between leisure and self-esteem for reasons of gender, culture and age and which subjective aspects of leisure are convergent with global self-esteem or with some of its dimensions.

Finally, if we are to deepen our understanding of the relationship between leisure and self-esteem, an important advance, apart from research where the participants structure information and the experiential and multidimensional approaches are valued, would be to develop research from a mixed perspective, i.e., with studies that combine the internal and external approach, as seen in one of the articles in this review [76]. This approach, which could be multi-method or mixed-methods, would constitute an important source of new findings, as shown by the leading role occupied by this methodological movement in recent years [56,108,109].

The systematic review presented in this article began with the analysis of one of the databases most used by researchers, i.e., one of the most important indexes of research trends. However, for a more complete and thorough analysis, it would be necessary to expand the sample with the results obtained from other scientific databases such as Scopus or PsycInfo. The analysis carried out also focused on “how” the relationship between leisure and self-esteem is studied; from which methodological and conceptual approaches. Nonetheless, studies with more applied goals should undoubtedly contemplate, for example, the valuations made about leisure activities in particular, the leisure experiences that most contribute to self-esteem, the differences based on gender, culture or age in the relationships between leisure and self-esteem, and the ages at which most attention is paid to the relationships between leisure and self-esteem. However, despite these limitations, this systematic review of the relationships between leisure and self-esteem reveals a hitherto unknown reality regarding the scientific fundamentals. It tests a series of frameworks to evaluate, locate and plan research work and reveal the dominant and minority approaches. It reflects on the possibilities and limits of different combinations, and it highlights those less explored and more promising approaches in terms of deepening the understanding of the relationships between these constructs.

## 5. Conclusions

With this systematic review, we bring to light some of the challenges facing research on leisure and self-esteem with regard to the attainment of specific knowledge, which has an impact on intervention strategies and programs in this field. In view of the results, it should be noted that the relationship between leisure and self-esteem research requires further research. In other words, there is a general need for more studies that rigorously assess the relationship between leisure and self-esteem, a relationship that is confirmed in numerous papers but to which only a few (49 since 1992) give any degree of centrality and work on empirically. In particular, more studies are needed to cover the different approaches to the analysis of leisure and self-esteem. In effect, the reviewed articles show that the observational approaches are the most dominant, which, although they provide important information, fail to register the participants’ points of view. Thus, with respect to leisure, there is a need for further investigation of what leisure activities and experiences are relevant to individuals and consequently significant for their self-esteem, there also being a need to record which aspects or areas of activity most affect it. The predominant trends in the study of this relationship lead us to conclude that the relationship between leisure and self-esteem is indeed documented and supported in various scientific works, but since there is very little pluralism, many nuances of this relationship remain unidentified; this is a relationship that is possibly much deeper and wide-ranging than what has been documented. In short, professionals involved in research on leisure and self-esteem face the challenge of revaluing this relationship. To obtain a comprehensive understanding of this topic, they need to take into account the different approaches, while continuing to attend to more practical aspects of the differences in this relationship (gender, age or culture, for example) and the implications of this relationship on individual wellbeing, a circumstance that depends on both leisure and self-esteem. In any case, this study shows, on the one hand, that the relationship between leisure and self-esteem is only partially understood and, on the other hand, it shows that it is necessary and possible to advance in this understanding by using the pluralism of conceptual and methodological approaches that serve to explore this relationship. In this respect, frameworks of reference are provided as a means of organizing, guiding and reflecting on the possibilities and limits of the different approaches. Furthermore, an assessment is made of those approaches that currently make a greater contribution to the understanding of the relationships between leisure and self-esteem.

## Figures and Tables

**Table 1:** Research approaches for defining and measuring leisure [16] (p. 58). (adapted from: Kleiber, Walker and Mannell [18]).

	External	Internal
Objective	Behavioral-Observer	Behavioral-Participant
Subjective	Experiential-Observer	Experiential-Participant

**Table 2:** Research approaches in the conception and investigation of self-esteem (prepared by the authors).

	External	Internal
Unidimensional	Unidimensional-Observer	Unidimensional-Participant
Multidimensional	Multidimensional-Observer	Multidimensional-Participant

**Table 3:** Articles selected.

Article	Research Approaches to Defining and Measuring Leisure	Leisure Instrument	Research Approaches to Defining and Measuring Self-Esteem	Self-Esteem Instrument
[57]	Behavioral-experiential-participant	Interview	Unidimensional-participant	Semi-structured interview
[58]	Experiential-observer	Leisure Attitude Scale (Short Version), Leisure Satisfaction Scale	Unidimensional-observer	RSES *
[59]	Behavioral-observer	Ad hoc questionnaire	Unidimensional-observer	RSES
[60]	Behavioral-observer	Ad hoc questionnaire	Unidimensional-observer	RSES
[61]	Experiential-observer	Panel Survey of Employment for the Disabled (PSED, 2012)	Unidimensional-observer	RSES
[9]	Experiential-observer	Meaning, Global Leisure Meanings (2016)	Unidimensional-observer	RSES
[62]	Behavioral-observer	Ad hoc questionnaire	Unidimensional-observer	Dutch Personality Inventory
[63]	Behavioral-observer	Ad hoc questionnaire	Unidimensional-observer	RSES
[64]	Behavioral-observer	Seok Questionnaire (2005)	Unidimensional-observer	RSES
[65]	Behavioral-observer	Lawton Instrumental Activities of Daily Living Scale (IADL)	Unidimensional-observer	RSES
[66]	Behavioral-observer and Experiential-observer	Children’s Assessment of Participation and Enjoyment (CAPE) and Preferences for Activities of Children (PAC)	Multidimensional-observer	Piers-Harris Self Concept Scale (Piers Harris-2)
[67]	Behavioral-observer and Experiential-observer	Leisure Participation Survey (SWBIRS) and Leisure Satisfaction Scale (1980)	Unidimensional-observer	RSES
[68]	Behavioral-observer	Semi-structured interview	Unidimensional-observer	RSES
[69]	Behavioral-observer and Experiential-observer	Ad hoc: index of sport activity and items measuring the self-efficacy, competence and attractiveness in the physical domain	Multidimensional-observer	RSES and Facets of the physical self-concept (2004)
[70]	Behavioral-observer	Gambling Activity Measurement Tool (GAMT)	Unidimensional-observer	RSES
[71]	Behavioral-observer	Leisure Activities Scale (1978)	Unidimensional-observer	RSES
[72]	Behavioral-observer	WHO (1995)	Unidimensional-observer	RSES
[73]	Behavioral-participant	Daily activities reported by the respondents	Unidimensional-observer	RSES
[74]	Behavioral-observer	Generalized Problematic Internet Use Scale (GPIUS, 2002)	Unidimensional-observer	RSES
[75]	Behavioral-participant	Open questions	Unidimensional-observer	Performance-Based Self-Esteem scale (PBSE)
[76]	Behavioral-observer	Client Assessment of Strengths Interests and Goals (CASIG, 2001)	Unidimensional-observer	Self-Esteem Rating Scale-Short-Form (SERS-SF, 2006)
[77]	Behavioral-observer	Questions about lifestyle: 1 item: leisure-time daily among friends	Unidimensional-observer	RSES
[78]	Behavioral-observer	Ad hoc questionnaire	Unidimensional-observer	RSES
[79]	Behavioral-observer and Behavioral-participant	Minnesota Leisure Time Physical Activity Questionnaire and Four open-ended questions to elicit additional activities	Unidimensional-observer	RSES
[80]	Experiential-observer	Quality of Life Enjoyment and Satisfaction Questionnaire (Q-LES-Q) and Lancashire Quality of Life Profile (LQOLP)	Unidimensional-observer	RSES
[81]	Experiential-observer	Quality of Life (QOL)	Unidimensional-observer	QOL
[82]	Experiential-participant	Ad hoc questionnaire	Unidimensional-observer	RSES
[83]	Experiential-observer	Social adjustment scale (SAS, 1976)	Unidimensional-observer	RSES
[84]	Behavioral-observer and Experiential-observer	Adolescent-perceived Microsystems Scales: Social support, daily hassles and involvement (1995)	Multidimensional-observer	Self-Perception Profile for Adolescents (1987)
[85]	Behavioral-observer and Experiential-observer	Quality of Life Enjoyment and Satisfaction Questionnaire (Q-LES-Q)	Unidimensional-observer	RSES
[86]	Experiential-observer	Meaningful Leisure Activities Questionnaire (1999)	Unidimensional-observer	Global Self-Worth subscale (1986) Adult Self-Perception Profile (ASPP)
[87]	Behavioral-observer and Experiential-observer	Ad hoc Questionnaire social and solitary leisure activities and Meaningful Leisure Activities Questionnaire (1999)	Unidimensional-observer	Global Self-Worth (1986) Adult Self-Perception Profile (ASPP)
[88]	Experiential-observer	Quality Life Interview (1988), Satisfaction with leisure activities	Unidimensional-observer	RSES
[89]	Behavioral-observer	Questionnaire about different leisure activities and places	Unidimensional-observer	RSES
[90]	Behavioral-observer	Uni ad hoc item	Unidimensional-observer	RSES
[91]	Behavioral-observer and Experiential-observer	Participation and evaluation	Multidimensional-observer	Self-Perception Profile for Adolescents (1987)
[92]	Behavioral-observer	Social Adjustment Scale (SAS)	Unidimensional-observer	RSES
[93]	Experiential-observer	Leisure Benefit Scale	Unidimensional-observer	Self-esteem = “feeling good about myself,”
[94]	Experiential-observer	Lancashire QOL (Quality of Life) Profile	Unidimensional-observer	Lancashire Quality of Life Profile
[95]	Behavioral-observer and Experiential-observer	Questions about amount of time and evaluations	Unidimensional-observer	RSES
[96]	Behavioral-observer and Experiential-observer	Items describing experiences about leisure in natural areas	Unidimensional-observer	Items for self-esteem adapted
[97]	Behavioral-observer and Experiential-observer	Adolescent Leisure-Time Use Inventory	Multidimensional-observer	Self-Rating Scale (SRS, 1984)
[98]	Behavioral-participant	Request to list non work activities	Unidimensional-observer	Inventory used by Meir, Melamed and Abu-Freha (1990).
[99]	Behavioral-observer and Experiential-observer	Ad hoc instrument: frequency, duration and feelings	Multidimensional-observer	Batlle’s Culture Free Self-esteem
[1]	Experiential-observer	Leisure Orientation Scale (LOS)	Unidimensional-observer	RSES
[100]	Behavioral-observer and Experiential-observer	Activity of Daily Living Scale (ADL, 1963) and Five Leisure Satisfaction Questions	Unidimensional-observer	RSES
[101]	Experiential-observer	Measure of Perceived constraints on leisure	Unidimensional-observer	RSES
[102]	Behavioral-observer	Ad hoc checklist	Unidimensional-observer	Coopersmith Inventory
[103]	Experiential-participant	Leisure satisfaction interview	Unidimensional-observer	RSES

* RSES = Rosenberg Self-Esteem Scale (1965).

**Table 4:** Research approaches in the study of the relationship between leisure and self-esteem.

	Definitional Vantage Point												Total
Leisure	Observer				Participant				Observer-Participant				
Self-Esteem	UD-E	UD-I	MD-E	MD-I	UD-E	UD-I	MD-E	MD-I	UD-E	UD-I	MD-E	MD-I	
Behavioral	18	0	0	0	3	0	0	0	1	0	0	0	22
Experiential	12	0	0	0	2	0	0	0	0	0	0	0	14
Behavioral-experiential	6	0	6	0	0	1	0	0	0	0	0	0	13
Total	36	0	6	0	5	1	0	0	1	0	0	0	49

UD = Unidimensional; MD = Multidimensional; E = External or observer; I = Internal or participant.
